# Quercetin Loaded Monolaurate Sugar Esters-Based Niosomes: Sustained Release and Mutual Antioxidant—Hepatoprotective Interplay

**DOI:** 10.3390/pharmaceutics12020143

**Published:** 2020-02-09

**Authors:** Enas Elmowafy, Marwa O. El-Derany, Francesca Biondo, Mattia Tiboni, Luca Casettari, Mahmoud E. Soliman

**Affiliations:** 1 Department of Pharmaceutics and Industrial Pharmacy, Faculty of Pharmacy, Ain Shams University, Monazzamet Elwehda Elafrikeya Street, P.O.B. 11566 Abbaseyya, Cairo, Egypt; enasmostafa@pharma.asu.edu.eg; 2 Department of Biochemistry, Faculty of Pharmacy, Ain Shams University, Monazzamet Elwehda Elafrikeya Street, P.O.B. 11566 Abbaseyya, Cairo, Egypt; marwa.omar@pharma.asu.edu.eg; 3 Department of Biomolecular Sciences, School of Pharmacy, University of Urbino, Piazza Rinascimento, 6, 61029 Urbino (PU), Italy; f.biondo1@campus.uniurb.it (F.B.); mattia.tiboni@uniurb.it (M.T.)

**Keywords:** glucose laurate, sucrose laurate, trehalose laurate, sorbitan laurate, quercetin, ethanol injection, niosomes, antioxidant effect, hepatoprotection

## Abstract

Flavonoids possess different interesting biological properties, including antibacterial, antiviral, anti-inflammatory and antioxidant activities. However, unfortunately, these molecules present different bottlenecks, such as low aqueous solubility, photo and oxidative degradability, high first-pass effect, poor intestinal absorption and, hence, low systemic bioavailability. A variety of delivery systems have been developed to circumvent these drawbacks, and among them, in this work niosomes have been selected to encapsulate the hepatoprotective natural flavonoid quercetin. The aim of this study was to prepare nanosized quercetin-loaded niosomes, formulated with different monolaurate sugar esters (i.e., sorbitan C12; glucose C12; trehalose C12; sucrose C12) that act as non-ionic surfactants and with cholesterol as stabilizer (1:1 and 2:1 ratio). Niosomes were characterized under the physicochemical, thermal and morphological points of view. Moreover, after the analyses of the in vitro biocompatibility and the drug-release profile, the hepatoprotective activity of the selected niosomes was evaluated in vivo, using the carbon tetrachloride (CCl_4_)-induced hepatotoxicity in rats. Furthermore, the levels of glutathione and glutathione peroxidase (GSH and GPX) were measured. Based on results, the best formulation selected was glucose laurate/cholesterol at molar ratio of 1:1, presenting spherical shape and a particle size (PS) of 161 ± 4.6 nm, with a drug encapsulation efficiency (EE%) as high as 83.6 ± 3.7% and sustained quercetin release. These niosomes showed higher hepatoprotective effect compared to free quercetin in vivo, measuring serum biomarker enzymes (i.e., alanine and aspartate transaminases (ALT and AST)) and serum biochemical parameters (i.e., alkaline phosphatase (ALP) and total proteins), while following the histopathological investigation. This study confirms the ability of quercetin loaded niosomes to reverse CCl_4_ intoxication and to carry out an antioxidant effect.

## 1. Introduction

Flavonoids are natural molecules highly found in herbs, vegetables and fruits. They possess different biological properties, including antibacterial, antiviral, antihistamine, anti-inflammatory and anticancer activities [1,2,3]. These intrinsic properties, together with their biocompatibility and remarkable antioxidant activity, have attracted researchers’ attention to explore potential therapeutic applications [4,5].

Quercetin (3, 30, 4, 5, 7-pentahydroxyflavone) is probably the most studied bioflavonoid, belonging to the family of flavonols; its therapeutic applications include hepatoprotection [6], prevention of neural cell apoptosis [7] and cancer chemo-prevention and treatment [8].

Quercetin acts as strong antioxidant by scavenging free radicals and transition metal ions, thus decreasing the process of lipid peroxidation, which is responsible for the development of many diseases, e.g., cardiovascular and neurodegenerative diseases, as well as liver damage [9,10,11].

However, quercetin therapeutic applications present challenges due to (i) low aqueous solubility, (ii) photo and oxidative degradability, (iii) high first-pass effect, (iv) poor intestinal absorption and, hence, low systemic bioavailability [12,13,14,15].

In order to overcome these problems, which are common among flavonoids, drug delivery systems (DDS) including emulsions, cyclodextrins, polymeric nanoparticles, micelles and liposomes have been used to enhance their pharmaceutical properties [16,17,18].

Out of various DDS, niosomes are similar to liposomes in structures, preparation techniques, and physical properties. Niosomes possessed many advantages, including (i) biocompatibility, (ii) improved stability, (iii) non-immunogenicity, (iv) sustained drug release and (v) low preparation cost [19,20,21,22].

Non-ionic surfactants, which present high safety and biocompatibility, have been selected over ionic surfactants for the preparation of these vesicles because anionic and cationic charges presented irritant and cytotoxic effects, respectively [23,24].

Non-ionic surfactants are comprised of both polar and nonpolar segments and possess high interfacial activity. The formation of bilayer vesicles instead of micelles is dependent on the hydrophilic–lipophilic balance (HLB). HLB values between 3 and 8 are compatible with the preparation of bilayer surfaces and refer to water-in-oil (W/O) emulsifiers [21,25].

Sugar esters are a class of non-ionic surfactants which are cheap, renewable and can be used in many applications, including solubilization, stabilization and emulsification, in food, cosmetic and pharmaceutical industries [26]. Being slightly hydrophilic (HLB 9-13), sugar esters form more stable niosomes when mixed with cholesterol or other hydrophobic surfactants [27].

Here, we aimed to formulate quercetin-loaded niosomes, using different sugar-based esters stabilized with cholesterol at different ratios. The prepared niosomes were firstly characterized for their physicochemical, thermal and morphology properties. Then, drug encapsulation efficiency was measured, and selected quercetin-loaded niosomes were evaluated for their biocompatibility, antioxidant and hepatoprotective properties, both in vitro and in vivo.

## 2. Materials and Methods

Glucose, lauric acid, quercetin (>95%), cholesterol, sucrose monolaurate, sorbitan monolaurate (Span^®^ 20), trimethylsilyl chloride, 4-(dimethylamino) pyridine (DMAP) and *N*,*N*′-dicyclohexylcarbodiimide (DCC), tween 80, carbon tetrachloride (CCl_4_) and 3-(4,5-dimethylthiazol-2-yl)-2,5-diphenyltetrazolium bromide (MTT) were purchased from Sigma-Aldrich (St. Louis, MO, USA) and used as received. Triethylamine was purchased from FluoroChem (Hadfield, UK). Trehalose was kindly donated by Asahi Kasei Chemical Corporation (Tokyo, Japan). All reactions were conducted under a dry nitrogen atmosphere. All organic solvents used in the study were purchased from Sigma-Aldrich, and prior to use, acetone was dried, using molecular sieves with an effective pore diameter of 4 Å. Column chromatography purifications were performed under “flash” conditions, using Merck 230–400 mesh silica gel. TLC was carried out on Merck silica gel 60 F254 plates, which were visualized by exposure to ultraviolet light and by exposure to an aqueous solution of ceric ammonium molybdate (CAM). The structures of compounds were characterized by ^1^H NMR techniques. Chemical shifts are given in ppm relative to the residual solvent peak as internal standard.

### 2.1. Synthesis of Sugar Esters

#### 2.1.1. Synthesis of Glucose Monolaurate


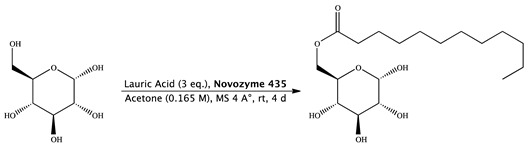


Novozyme 435 (0.168 g) was added to a solution of lauric acid (0.475 g, 1.68 mmol, 0.531 mL), molecular sieves (0.336 g) and d-(+)-glucose (0.100 g, 0.56 mmol) in 3.39 mL of acetone (0.165 M). The mixture was stirred at 25 °C for 4 days, diluted with acetone and filtered, and then the filtrate was concentrated. The purification of the residue was done by column chromatography (cyclohexane/ethyl acetate 6:4) and gave 6-*O*-monolauroyl-glucose as a white solid. (0.163 g, Yield: 80%) [28].

^1^H NMR (400 MHz, DMSO-*d*6) δ 6.33 (d, *J* = 4.5 Hz, 1H, OH^1^), 5.02 (d, *J* = 5.1 Hz, 1H, H^4^), 4.89 (dd, *J* = 3.8 Hz, 1H, H^1^), 4.74 (bs, 1H, OH^3^), 4.51 (d, *J* = 5.0 Hz, 1H, OH^2^), 4.26 (dd, *J* = 1.9, 11.6 Hz, 1H, H^6^), 3.99 (dd, *J* = 5.5, 17.9 Hz, 1H, H^6′^), 3.77 (ddd, *J* = 2.0, 6.2, 10.1, Hz, 1H, H^5^), 3.42 (dd, *J* = 9.2 Hz, 1H, H^3^), 3.12 (m, 1H, H^2^), 3.04 (ddd, *J* = 4.2, 9.2 Hz, 1H, H^4^), 1.98 (t, 2H, H^8^), 1.49 (m, 2H, H^9^), 1.33–1.19 (m, 16, –CH2– chain length oleic acid), 0.87–0.83 (t, *J* = 14.07, 3H, 18-H) ppm.

#### 2.1.2. Synthesis of Trehalose Monolaurate

Synthesis of 2,3,4,2′,3′,4′-hexakis-*O*-(trimethylsilyl)-α,α-trehalose (**3**):


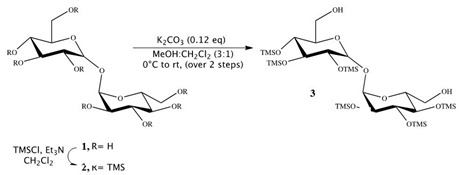


Triethylamine (18.3 mL, 130.9 mmol) was added to a stirred suspension of trehalose **1** (1.12 g, 3.27 mmol) in dichloromethane (15 mL). Reaction mixture was cooled to 0 °C, and trimethylsilyl chloride (4.87 mL, 39.26 mmol) was added to it. The solution was stirred at RT for 12 h, and then an additional 1.6 mL of trimethylsilyl chloride (13.08 mmol) was added at 0 °C, and the reaction was stirred for 4 h at RT. Solvents were then evaporated on rotor, and the crude product was extracted in petroleum ether (50 mL × 5). The combined organic layer was dried on anhydrous sodium sulfate and concentrated in vacuum to obtain 2,3,4,6,2′,3′,4′,6′-octakis-*O*-(trimethylsilyl)-α,α-trehalose **2** as a cream-yellow solid (3.04 g). Compound **2** showed no impurity peaks in ^1^H NMR spectrum [29,30,31].

^1^H NMR (400 MHz, CDCl3) δ 4.91 (d, *J* = 3.1 Hz, 2H), 3.88 (t, *J* = 8.8 Hz, 2H), 3.80–3.77 (m, 2H), 3.71–3.64 (m, 4H), 3.42 (t, *J* = 9.3 Hz, 2H), 3.39 (dd, *J* = 3.1, 9.3 Hz, 2H) 0.14 (s, 18H), 0.13 (s, 18H), 0.11 (s, 18H), 0.09 (s, 18H).

To a cooled solution of compound **2** (3.04 g, 3.31 mmol) in methanol and dichloromethane (19 mL, 3:1) at 0 °C, K_2_CO_3_ (54 mg, 0.40 mmol) was added and the reaction was stirred for 15 min at 0 °C and then at RT for 1 h. Reaction was quenched by addition of acetic acid (0.7 mL). Solvents were evaporated in vacuum, and crude product was separated by column chromatography on silica gel (2:8 ethyl acetate/petroleum ether) to afford compound **3** (2.38 g, Yield: 92%) as a white solid. Compound **3** showed no impurity peaks in ^1^H NMR spectrum. ^1^H NMR (400 MHz, CDCl3) δ 5.05 (d, *J* = 3.1 Hz, 2H, H-1, H-1′), 4.04 (t, *J* = 9.0 Hz, 2H, H-3, H-3′), 4.01 (dt, *J* = 3.1, 9.0 Hz, 2H, H-5, H-5′), 3.88–3.80 (m, 4H, H-6, H-6′), 3.63 (t, *J* = 9.0 Hz, 2H, H-4, H-4′), 3.57 (dd, *J* = 3.1, 9.0 Hz, 2H, H-2, H-2′), 1.97 (bt, 2H), 0.31 (s, 18H), 0.29 (s, 18H), 0.27 (s, 18H).

Synthesis of 6-*O*-monolauroyl-2,3,4,2′,2′,4′-hexakis-*O*-(trimethylsilyl)-α,α-trehalose (**4**):


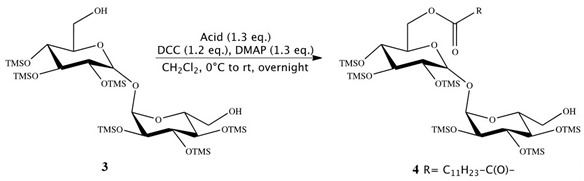


Then, 2, 3, 4, 2′, 3′,4′-Hexa-*O*-(trimethylsilyl)-α,α-trehalose (**3**) (0.770 g, 1.00 mmol), together with 4-DMAP (0.160 g, 1.30 mmol) and lauric acid (C_12_H_24_O_2_) (1.30 mmol), was placed in a flame-dried, 25 mL Schlenk flask, containing anhydrous dichloromethane (5 mL). A solution of 1,3-DCC (0.250 g, 1.20 mmol) in anhydrous dichloromethane (3 mL) was added dropwise. The mixture was mechanically stirred overnight at room temperature, under a nitrogen atmosphere. The resulting precipitate was removed by filtration under reduced pressure and washed with dichloromethane (5 mL). The crude product was obtained by removing the solvent from the combined, dried dichloromethane solutions by flash evaporation and purified by flash column chromatography (cyclohexane, diethyl ether 7:3). Compound **4** showed no impurity peaks in ^1^H NMR spectrum. ^1^H NMR (CDCl3): d (ppm) = 4.92 (t, 2H, *J* = 3.2 Hz, 1-H, 1′-H), 4.29 (dd, 1H, *J*_1_ = 11.9 Hz, *J*_2_ = 2.5 Hz, 6-H), 4.06 (dd, 1H, *J*_1_ = 11.9 Hz, *J*_2_ = 4.7 Hz, 6-H), 4.01 (m, 1H, 5-H), 3.91 (m, 2H, 2-H, 3-H), 3.85 (m, 1H, 5′-H), 3.70 (m, 2H, 6′-H), 3.45 (m, 4H, 4-H, 2′-H, 3′-H, 4′-H), 2.35 (m, 2H, 2″-H), 1.63 (m, 2H, 3″-H), 1.26 (m, 16H, 4″-H, 11″-H), 0.88 (m, 3H, 12″-H), 0.14–0.10 (m, 54H, 6 SiMe3).

Synthesis of 6-*O*-monolauroyl-α,α-trehalose (**5**):


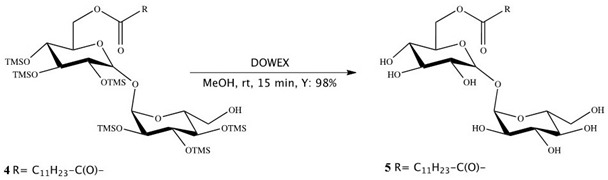


Dowex-H^+^ (10% by weight) was added to a solution of **4** (0.1 mmol) in dichloromethane/methanol (0.5 mL, 3:1, *v*/*v*), and the reaction mixture was stirred at RT. After 30 min, the mixture was filtered and concentrated in vacuum, and the resulting residue was crystallized to obtain the fully deprotected compound **5** in 97–98% yields. Compound **5** showed no impurity peaks in ^1^H NMR spectrum. ^1^H NMR (DMSO): d (ppm) = 5.08 (dd, 2H, *J*_1_ = 11.1 Hz, *J*_2_ = 3.6 Hz, 1-H, 1′-H), 4.36 (d, 1H, *J* = 11.7 Hz, 6-H), 4.19 (dd, 1H, *J*_1_ = 11.9 Hz, *J*_2_ = 4.9 Hz, 6-H), 4.02 (m, 1H, 5-H), 3.79 (m, 4H, 2-H, 2′-H, 5′-H, 6′-H), 3.66 (m, 1H, 6′-H), 3.47 (m, 2H, 3-H, 3′-H), 3.33 (m, 2H, 4-H, 4′-H), 2.34 (t, 2H, *J* = 7.2 Hz, 2″-H), 1.61 (m, 2H, 3″-H), 1.29 (m, 16H, 4″-H, 11″-H), 0.90 (t, 3H, *J* = 6.6 Hz, 12″-H).

### 2.2. Preparation of Niosomes

Accurately weighed amounts of the selected surfactant (Table 1) and cholesterol, in the molar ratios of 1:1 and 2:1, were dissolved in 5 mL of ethanol. Then, quercetin (10 mg) was added to surfactant–cholesterol solution. Deionized water (10 mL) was heated to 60 °C in a beaker, using heating magnetic stirrer. Then, quercetin/surfactant–cholesterol solution was dropped at slow rate on the hot deionized water, using a syringe, under stirring at 800 rpm. Ethanol was left to evaporate, with stirring for 2 h, and remaining traces of ethanol were removed by using a rotary evaporator at 60 °C. The formed niosomes appeared to be milky yellow. The selection of preparation temperature (60 °C) was to exceed the gel–liquid transition temperature (Tc) of tested surfactants. The niosomal dispersion volume was adjusted to 10 mL, using deionized water, and then left overnight at 4 °C, in the refrigerator, to mature before doing any further studies.

The same method was used to prepare plain niosomes without adding quercetin to the ethanolic solution.

### 2.3. Determination of Encapsulation Efficiency (EE%)

Encapsulation efficiency (EE%) for quercetin loaded niosomes was calculated by measuring the free quercetin concentration in the niosomal dispersion. The unencapsulated drug was separated by centrifugal filters (Nanosep^®^ centrifuge tube) with Mw cutoff 100 kDa. Then, 0.5 mL samples of the prepared niosomes were transferred to the upper chamber of the Nanosep^®^ and then centrifuged for 120 min, at 6000 rpm. The amount of unencapsulated quercetin in the collecting chamber liquid was measured by measuring the absorbance at 370 nm, using UV–vis spectrophotometer (Model UV–1601PC; Shimadzu, Kyoto, Japan), as previously described [33].

The EE (%) was calculated by using the following equation:

$$\mathrm{EE}\left(\%\right)=\frac{{\left[Drug\right]}_{total}-{\left[Drug\right]}_{free}}{{\left[Drug\right]}_{total}}\;\times 100$$

where [*Drug*]*_total_* is the total amount of added drug, and [*Drug*]*_free_* is the amount of unencapsulated free drug.

### 2.4. Size and Zeta-Potential Measurements

The average particle size (PS), polydispersity index (PDI) and zeta-potential (ZP) values of the quercetin-loaded niosomes were assessed by using dynamic light scattering (DLS), using a Nanosizer ZS series (Malvern Instruments, Southborough, MA, USA). Niosomes were diluted in deionized water and measurements were performed at 25 °C, using disposable polystyrene cells and plain folded capillary zeta cells for average size and zeta-potential values, respectively. The hydrodynamic diameters were measured and presented as the average value of 20 runs, with triplicate measurements for each run. Electrophoretic mobilities were measured and utilized to calculate the zeta-potential values, using the Helmholtz–Smoluchowski approach [34].

### 2.5. In Vitro Release Profile

Sample of the selected best formulation containing an amount of niosomes equivalent to 2.5 mg quercetin was put in a dialysis bag (Mw cutoff 10 kDa). The dialysis bag was then placed in 40 mL of phosphate buffered saline (PBS), pH 7.4, containing Tween 80 (1% *w*/*v*). Release was conducted at 37 °C, in a shaking water bath, at shaking velocity of 100 strokes/min. Then, 1.5 mL samples were removed from release medium at predetermined time intervals and replaced by the addition of an equal volume of prewarmed release medium, to maintain sink conditions during release experiment. The amount of drug released at different intervals was followed by measuring quercetin absorbance at 370 nm, using a UV/vis spectrophotometer. Drug release of 2.5 mg of quercetin in ethanol was done as a control experiment for comparison with release from niosomes. This control was also done to remove the effect of nonspecific adsorption of the drug to the dialysis membrane.

### 2.6. Differential Scanning Calorimetry

The thermal properties were estimated, using DSC (Shimadzu-DSC 60, Kyoto, Japan), at a heating rate of 10 °C/min, over a temperature range of 25–300 °C, using dry nitrogen as carrier gas, with a flow rate of 25 mL/min.

### 2.7. Fourier Transform Infrared (FT-IR) Spectroscopy

FT-IR spectra were recorded in the range of 4000–400 cm^−1^ on a Nicolet 6700 FT-IR (Thermo Scientific, Waltham, MA, USA). Powdered samples were loaded on KBr discs without special treatment. All spectra were recorded at a resolution of 4 cm^−1^ and 16 scans at ambient temperature [35].

### 2.8. Morphology of Niosomes

Morphological examination of the selected niosomes was performed by using a high-resolution transmission electron microscope (HR-TEM) (Jeol Electron Microscope, JEM-1010, Tokyo, Japan). A droplet of the dispersion was added to a carbon-film-covered copper grid, without staining. Excess liquid was blotted, using a filter paper, and then air-dried before analysis. Samples were visualized under electron beam at a voltage of 200 kV and magnification power ×30,000.

### 2.9. Physical Stability Study

Stability of the selected niosomes was determined by storing them at 4 ± 0.5 °C. Particle size (PS), Drug EE% and PDI were monitored after 14 and 30 days.

### 2.10. In Vitro Protective Effect of Quercetin Niosomes

#### 2.10.1. Cell Viability Assay

Human hepatocellular carcinoma (HepG2) cells (ATCC^®^ HB-8065^TM^) were obtained from VACSERA (Giza, Egypt). HepG2 cells were cultured in Roswell Park Memorial Institute (RPMI) medium with 10% fetal bovine serum (FBS), 2 mM l-glutamine and 2 mM penicillin, streptomycin at 37 °C in atmosphere of 5% CO_2_ in a humidified incubator. HepG2 cells were seeded in 96-well plates (10^4^ cells/well) and allowed to attach to the well surface for 24 h, at 37 °C, in a 5% CO_2_ humidified atmosphere. Free quercetin (drug concentrations equivalent to 1, 2.5, 5, 10, 20 µg/mL), quercetin–niosomes (with drug equivalent concentrations 1, 2.5, 5, 10, 20 µg/mL) and plain noisomes (concentrations 5, 12.5, 25, 50, 100, 200, 400 µg/mL) were added to the cells and incubated for 24 h. For each sample, a set of at least 6 wells was used. After the treatment, the culture medium was replaced by medium containing MTT solution (5 mg/mL in phosphate buffer saline (PBS)). Then, after 4 h of incubation, intracellularly formed formazan crystals were dissolved by addition of 50 µL of DMSO and absorbance was measured in a microplate reader at 600 nm (Chromate, Bohemia, NY, USA).

#### 2.10.2. Protective Effect on H_2_O_2_-Induced Oxidative Stress in HepG2 Cells

In the oxidative stress paradigm, cells (10^4^ cells/well) were exposed to 0.1 mM H_2_O_2_ for 1 h, to obtain submaximal cytotoxicity. Cells were pretreated for 1 h with free quercetin or quercetin–niosomes (drug concentration equivalent to 10 µg/mL) and plain niosomes (50 µg/mL) before the H_2_O_2_ pulse, to evaluate the protective effect. Viability was assayed after 24 h by MTT-dye reduction assay.

### 2.11. In Vivo Studies

#### 2.11.1. CCl_4_-Induced Hepatotoxicity

To determine the hepatoprotective potential of quercetin-loaded niosomes, serum biomarker enzymes (ALT and AST) and biochemical parameters (ALP and total protein) were demonstrated via chemically inducing acute liver injury in vivo. The protocol followed for the experiment was approved by the Research Ethics Committee of the Faculty of Pharmacy, Ain Shams University (Cairo, Egypt: ENREC-ASU-2020-66). The experiment was conducted over 6 days. Twenty-four healthy male albino rats (150–200 g) were divided into four groups of six animals each, as follows:

- Group I: a negative control group that received normal saline;
- Group II: a positive control group that received a single dose of CCl_4_ in corn oil (1:1 *v*/*v*), injected intraperitoneally, at a dose of 1 mL/kg on the fifth day;
- Group III: received free quercetin in normal saline solution daily at a dose of 30 mg/kg [36];
- Group IV: received optimum quercetin–niosomes (containing an equivalent concentration of 30 mg/kg of quercetin).

Intraperitoneal injection was performed in groups III and IV, daily, for five days. Then, these groups were injected intraperitoneally with a single dose of CCl_4_ on the fifth day of the study, 1 h after the injection of the last dose.

#### 2.11.2. Assessment of Oxidative Stress Parameters

The rat livers were extracted, washed out with cold PBS, weighted and homogenized with appropriate buffer. The tissue homogenate was centrifuged at 4000 rpm for 20 min. The supernatant was used directly to determine the amount of reduced GSH; however, GSH peroxidase (GPX) activity was determined by a kinetic method, using a commercial kit (Biodiagnostic, Cairo, Egypt).

#### 2.11.3. Histopathological Assessment of Liver Tissues

At the end of a six-day period, after dosing, the animals were decapitated. Liver specimens were collected and fixed in 10% *w*/*v* formalin solution for 24 h. Then, embedding in paraffin bees wax tissue blocks at 4 μm thickness was performed, using sledge microtome (Rotary Leica RM2245, Vista, CA, USA). The collected sample sections were deparaffinized, stained by hematoxylin and eosin and assessed for severity of liver injury via histopathological microscopical imaging, at 10× magnifications.

### 2.12. Statistical Analysis

Data presented in this study were expressed as mean of 3 or 6 independent experiments ± standard deviation (SD) or standard error of mean (SEM). The data obtained were compared by using one-way analysis of variance (ANOVA), and the significance of difference between formulations was calculated by Tukey–Kramer multiple comparison, using Graph Pad Instat^®^ software (GraphPad Software, La Jolla, CA, USA).

## 3. Results and Discussion

The present investigation was designed to demonstrate the vesicle-forming potential of novel sugar esters as biosurfactants, namely glucose C12, sucrose C12 and trehalose C12, for the manufacture of quercetin-loaded nanocarrier. A member of the Span^®^ family, specifically Span^®^ 20 “sorbitan monolaurate”, was also tried for niosomes preparation with comparative purposes. Two cholesterol: non-ionic surfactant molar ratios were tested (1:1 and 2:1) to study their effect on the physicochemical properties of niosomes. Ethanol injection technique was adopted due to its well-ascribed simplicity and safety [37]. Among different utilized organic solvents, ethanol was found to be a good solvent for niosomes components, including quercetin, avoiding any aggregation during preparation.

### 3.1. Preparation and Characterization of Quercetin Loaded Niosomes

As shown in Table 2, quercetin-loaded niosomes were successfully prepared and characterized, by measuring their PS, PDI, ZP and EE%. The sugar esters surfactants used in the preparation of quercetin loaded niosomes have different HLB values (glucose C12 = 9.89, sucrose C12 = 13.01 and trehalose C12 = 13.01). Span^®^ 20 (HLB = 8.6) was used to prepare niosomes for comparison, having the same length of hydrocarbon chain of sugar lauroyl esters.

The PS of prepared niosomes were in the range of 161.0 ± 4.61–224.4 ± 4.38 nm. Among the different studied surfactants, glucose C12 had the smallest recorded size (F2). This might be due to higher hydrophobic–hydrophobic interaction between cholesterol, hydrophobic chain of glucose C12 and encapsulated hydrophobic quercetin as compared to lower interaction in case of surfactants with higher hydrophilicity [20].

It is worthy to note that the PS of niosomes prepared at surfactant:cholesterol ratio 2:1 were significantly higher than niosomes prepared at the ratio 1:1, using the same surfactants. This was in agreement with previous studies, which show that increasing cholesterol content in niosomes increase their stability [38] and decrease their size [39,40]. Furthermore, it can be observed that there were no significant differences between the niosomes prepared using different surfactants when prepared at surfactant:cholesterol ratio of 1:1 (*p* > 0.05). However, when the surfactant quantity increased (i.e., surfactant:cholesterol ratio of 2:1) the effect of surfactant HLB on niosome PS was obvious. The PS of niosomes was found to increase with the increase of HLB of used surfactant. This could be due to the fact that the more hydrophilic the surfactant used in the niosomal formulation, the larger the vesicles formed [38].

With the exception of F7 and F8, all the niosomes showed small and acceptable PDI values (less than 0.25). Moreover, the ZP values of niosomes were negative, with values within acceptable range, between −35 and −25 mV, indicating the formation of stable niosomes [40]. The negative charge carried by the vesicles could be due to the charge carried by the drug at the pH of the dispersion. The measured EE% was found to be higher than 55%. The highest measured entrapment values of 83.59% and 80.85% were obtained for F2 and F1, made of glucose C12 and Span^®^ 20, respectively. Interestingly, there was no statistical difference between both formulae (*p* > 0.05). This could be attributed to the influence of HLB values of both span^®^ 20 and glucose C12 (HLB values of 8.6 and 9.89, respectively) that heightened the hydrophobicity of the bilayer domain, greatly loading higher amount of hydrophobic quercetin [41]. Increasing the hydrophilicity of the other surfactants negatively affected the encapsulation efficiency of quercetin. Unlike its effect on vesicles size, increasing molar ratio led to an obvious decrease in quercetin-loading inside niosomes. Increasing the number of surfactants and keeping the amount of cholesterol constant might decrease the bilayer rigidity, promoting drug-leakage-form niosomes. This finding was consistent with what was stated in previous studies [38].

Combining low size and the highest drug encapsulation, niosomes formed of glucose monolaurate/cholesterol in a ratio of 1:1 (F2) were selected for further studies.

### 3.2. In Vitro Drug Release

**Figure 1:**
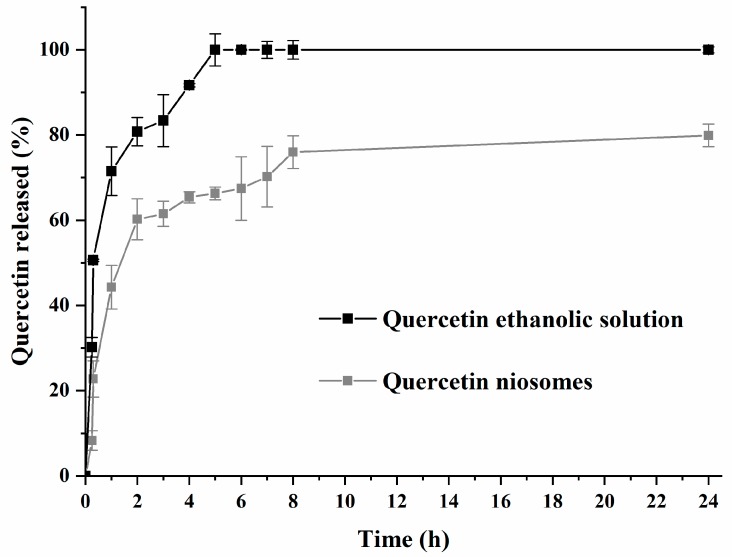
Release profile of quercetin-loaded niosomes (F2) and free drug (quercetin ethanolic solution).

Figure 1 shows drug release from an ethanolic solution and from niosomes (F2 formula). The initial burst release (44.29% after 1 h) was observed for quercetin-loaded niosomes. After 24 h, approximately 79.9% of entrapped quercetin was released from niosomes, while quercetin was released completely from ethanolic solution, over a period of 24 h (positive control). This indicated sustained release pattern of quercetin loaded niosomes. A burst-release effect was initially observed because of the rapid efflux of the free drug or that associated with the outer surface of the niosomes. This was followed by a slow diffusion from the bilayer of the membrane forming the niosomes, showing significant statistical difference in quercetin release when compared to ethanolic quercetin solution (*p* < 0.01). In addition, the presence of cholesterol, as well as the long hydrophobic alkyl chains (C12) of the tested surfactant, leads to the formation of compact niosomes, which retard the rate of drug release [42]. Finally, the quercetin-release kinetic model from niosomes was best fitted by Higuchi, with an R^2^ value of 0.9389, indicating that the release mechanism is diffusion controlled. This was consistent with previous findings reporting the diffusion-release mechanism of niosomes [41].

### 3.3. TEM Imaging of Niosomes

**Figure 2:**
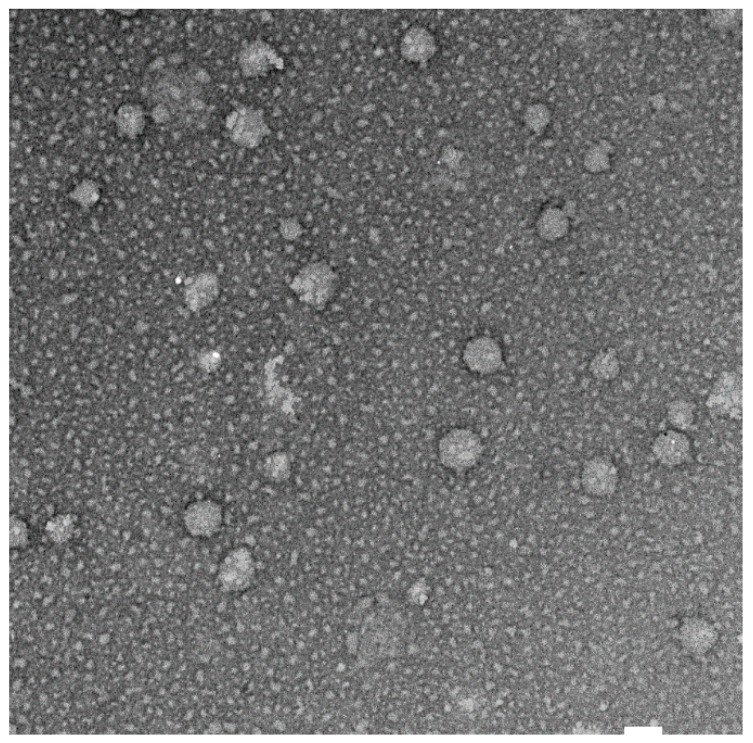
TEM image of quercetin loaded niosomes (F2) at magnification of ×30,000, white bar 100 nm.

The TEM image confirmed the formation of quercetin-loaded niosomes (Figure 2) and showed the spherical structure and monodisperse size distribution of the formed vesicles with a similar appearance to the typical shape of images captured in previous studies [43]. In addition, the vesicles’ average size obtained from TEM images (140.4  ±  25.2 nm) was similar but slightly smaller to that measured by DLS technique (161.0 ± 4.61 nm) due to the drying effect that happened during sample preparation. Moreover, the particle-size distribution showed monomodal size distribution of F2 formulation.

### 3.4. DSC Studies

**Figure 3:**
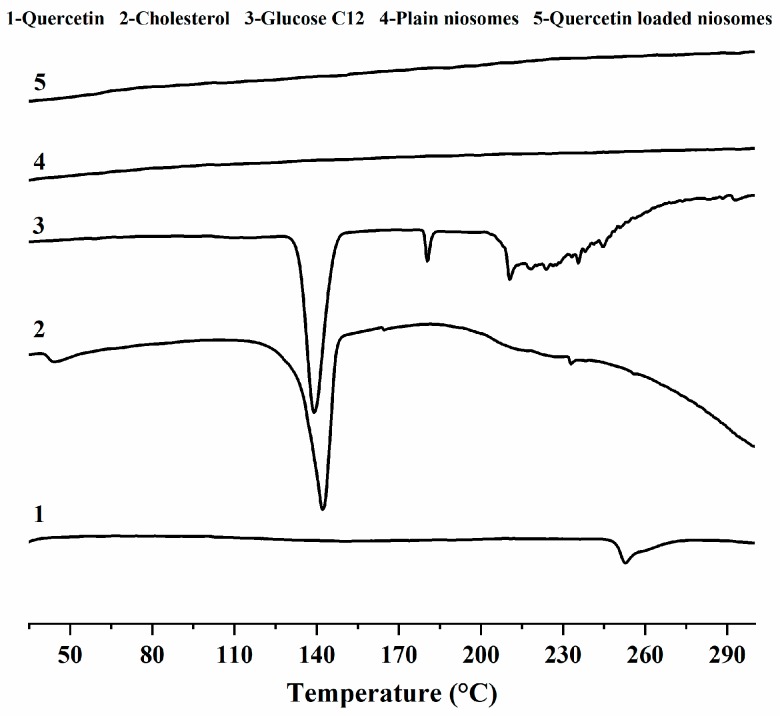
DSC thermograms of quercetin (1), cholesterol (2), glucose monolaurate (3), plain niosomes (4) and quercetin loaded niosomes (5).

DSC thermograms of quercetin, cholesterol, glucose C12, plain niosomes and quercetin-loaded niosomes (F2) are shown in Figure 3. Quercetin showed a broad endothermic peak at 252.9 °C, with heat enthalpy of 17.7 J/g. This result was in accordance with those of previous studies [44].

The thermograms of cholesterol and glucose C12 demonstrates that they melt at 147 and 180.4 °C, with fusion enthalpies of 1.5 and 10.1 J/g, respectively.

Regarding the spectra of both plain and medicated niosomes, we observed the absence of the peaks corresponding to both niosome ingredients (cholesterol, glucose C12) and the drug (quercetin), indicating the good interaction between quercetin and niosomal ingredients, which cause bilayer rigidity and the loss of drug crystallinity [43]. The previous finding also shows the compatibility of niosomes with quercetin, and also interprets the high encapsulation and sustainment of drug release observed with F2.

### 3.5. FT-IR Studies

The FT-IR spectra of quercetin exhibited its distinguishing characteristic peaks at 3289.58 cm^−1^ (–OH stretching), 1672.73 cm^−1^ (C=O stretching), 1521.9 cm^−1^ (aromatic C=C stretching) and 1212 cm^−1^ (aromatic C–O stretching) [45,46]. Cholesterol showed its characteristic peaks at 3405.24 cm^−1^ (–OH stretching), 2866.69 cm^−1^ (CH_2_ symmetric stretching vibration), 1466.54 cm^−1^ (asymmetric stretching vibration of CH_2_ and CH_3_), 1376.82 cm^−1^ (CH_2_ and CH_3_ bending vibration) and 1056.58 cm^−1^ (C–O bending vibration) [47,48].

The FT-IR spectra of lauroyl glucose ester displayed the vibrations absorbance of glucose –OH group at 3331 cm^−1^, C–H of methylene groups at 2920.94 and 2850.35 cm^−1^, C=O ester at 1731.18 cm^−1^, C–O ester at 1156.32 cm^−1^ and C–O of glucose –OH at 1050.73 cm^−1^ [49].

As for plain niosomes spectrum, remarkable band-shifts and lowered intensities were observed. OH stretching peaks of cholesterol and glucose became broader, and their frequency positions were changed to 3416.37 cm^−1^. In addition, glucose ester stretching peaks were diminished, and their intensities were greatly lowered, concluding the successful niosomes formation [50].

**Figure 4:**
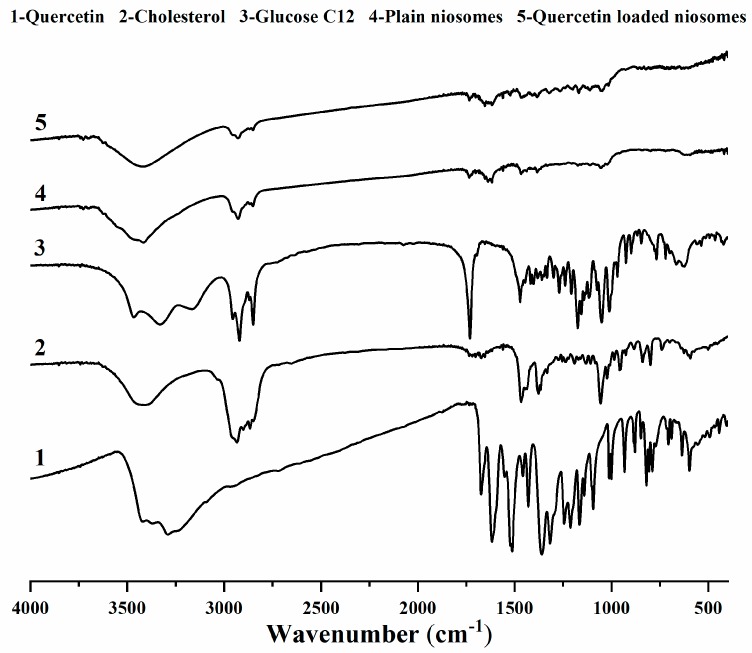
FTIR spectra of quercetin (1), cholesterol (2), glucose monolaurate (3), plain niosomes (4) and quercetin-loaded niosomes (5).

After the incorporation of quercetin in niosomes, a similar FT-IR spectrum of quercetin-loaded niosomes was obtained when compared to plain niosomes. The characteristic quercetin-related peaks were not identifiable, which could provide evidence for the possible quercetin–niosomes interaction, highlighting drug retention inside niosomes and its sustained release [51] (Figure 4).

### 3.6. Stability Studies

**Figure 5:**
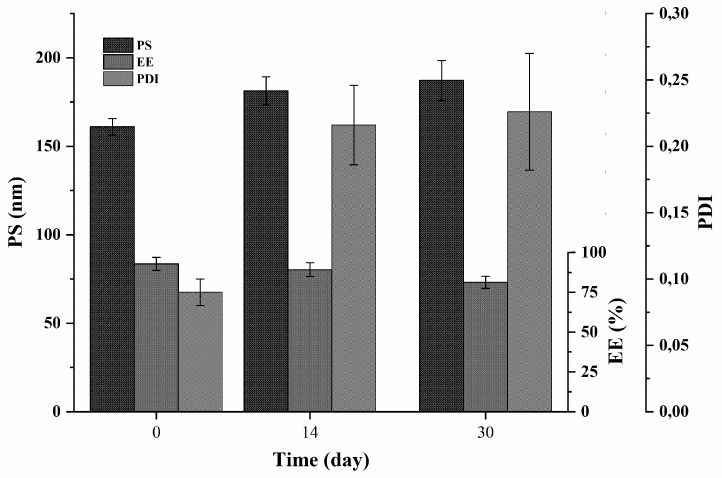
Physical stability (PS, PDI and EE (%)) of quercetin-loaded niosomes (F2).

The stability of the selected formulation (F2) was evaluated by following the changes in PS, PDI and EE (%) of quercetin-loaded niosomes (F2) during its storage at 4 °C for one month (Figure 5). It is worth to say that swelling or disruption of niosomes accompanied with drug leakage during their storage have been previously reported to occur [20]. As shown in Figure 5, the values of niosomal size and PDI increased significantly (*p* < 0.05) from the preparation time, when compared to the recorded values after 14 days; however, both PS and PDI only slightly changed, showing no statistical difference as the storage time increases to 30 days when compared to the values measured after two weeks, yet the recorded values of both PS and PDI after one month were within acceptable limits.

As for the encapsulation efficiency, a slight, non-significant leakage of the amount of encapsulated quercetin was noticed after 14 days. However, the drug was found to significantly escape from F2 niosomes when the drug entrapment was checked at the end of the experiment and compared to drug entrapment of freshly prepared niosomes. The percent drug leakage was approximately 12% at the end of 30 days as compared to the initial entrapment.

It is worth noting that the physical stability was performed only at refrigerator temperature, as we found that storing the niosomes at room temperature was proven to negatively affect their stability, due to lowered hydrophobic part rigidity at higher temperature [52].

### 3.7. In Vitro Protective Effect of Quercetin Niosomes

#### 3.7.1. Cell Viability Assay

**Figure 6:**
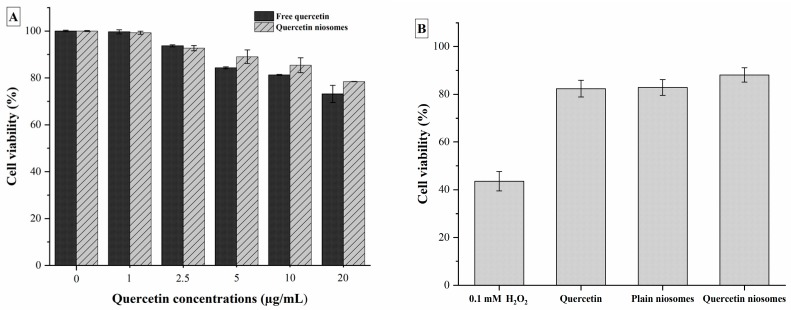
(**A**) Cell viability of HepG2 cells after exposure to quercetin and quercetin-loaded niosomes. (**B**) Protective effect on H_2_O_2_-induced oxidative stress in HepG2 cells after exposure to quercetin, plain and quercetin-loaded niosomes.

Cytotoxicity of quercetin, the selected formula (F2) and its corresponding plain niosomes (Figure 6A) was evaluated using MTT assay. HepG2 cells, as representative of liver cells, were widely utilized in assessment of nanocarriers’ safety, owing to its environmental-responsive morphology. The plain niosomes, made of cholesterol and glucose C12, were found to be safe on HepG2 cells up to a concentration of 400 µg/mL (data not shown), owing to the biocompatibility of the used niosomal components. Similarly, both free quercetin and loaded niosomes exhibited no toxicity against HepG2 cells.

#### 3.7.2. Protective Effect on H_2_O_2_-Induced Oxidative Stress in HepG2 Cells

The protective effect of free quercetin and quercetin niosomes against H_2_O_2_-induced oxidative stress in HepG2 cells was demonstrated. The viability of the cells treated with 0.1 mM H_2_O_2_ (1 h) was found to be significantly decreased by 56.48% compared to the untreated normal cells (*p* < 0.05). Pretreatment of HepG2 cells with free drug increased the cell survival by 48.01% (vs. H_2_O_2_ treatment). Higher significant protection was observed with quercetin niosomes, i.e., 50.6% increase in cell viability in comparison with H_2_O_2_ treatment (*p* < 0.05). Interestingly, plain niosomes exhibited a similar protective efficacy, increasing the cell viability (47.46% increase vs. H_2_O_2_ treatment) (Figure 6B). This can be due to the fact that glucose C12 is an ester of lauric acid, the main component of coconut oil, which is well-ascribed of possessing several biological activities, including antioxidant effect [49,53].

### 3.8. In Vivo Studies

#### 3.8.1. CCl_4_-Induced Hepatotoxicity

Carbon tetrachloride (CCl_4_) is well-known to cause serious hepatic toxicity, as manifested by liver necrosis and hepatic tissues steatosis, via the formation of various free radicals (trichloromethyl radical (CCl_3_) and trichloromethyl peroxyl radical (CCl_3_O_2_) [54].

It is worth mentioning that Ogundajo and his co-workers confirmed the greater capacity of parenteral quercetin to promote liver repair, minimizing liver degeneration, while maximizing antioxidant defense mechanism of the body, when compared with oral administration. In light, quercetin was injected intraperitoneally in this study, to maximize its effect, as well as decreasing its required dose [14].

Activities of both ALT and AST are well-monitored as hepatotoxicity biomarkers, evaluating eventual hepatic impairment. Both ALT and AST are present in the cytoplasm of the normal hepatocytes. However, leakage of these enzymes out into the blood occurs following liver damage or injury. As revealed in Table 3, compared with the normal control group, the activities of ALT and AST in the positive control CCL_4_ treated group were drastically elevated by approximately 4.39- and 4.00-fold, respectively (*p* < 0.05). This confirmed the severe cellular damage induced by CCl_4_ challenge. On the other hand, leakage of serum biomarker enzymes was effectively declined upon pretreatment with quercetin niosomes for five days, showing % reductions of 61.27% and 49.09% of values of ALT and AST, respectively. Percentage reductions of both ALT and AST were greatly lowered in the rat group pretreated with free quercetin (42.38% and 37.72%, respectively). Similarly, a significant increase in ALP and total protein values was detected in CCl_4_-treated group (1.28- and 1.11-fold increase compared to the normal untreated group, respectively). However, these parameters were normalized in the group that received quercetin niosomes for five days prior to CCl_4_ intoxication. For comparison, free quercetin effect on these latter parameters was also prominent and close to their normal values. Therefore, these results highlight the protective potential of the proposed quercetin niosomes against CCl_4_ hepatotoxicity and their superiority over free quercetin.

#### 3.8.2. Assessment of Oxidative Stress Parameters

Glutathione (GSH) is a major cellular free-radical-scavenging molecule that acts by maintaining cellular redox balance and, hence, protecting cells from reactive oxygen species (ROS) stress. Specifically, GSH is well-reported to covalently bind CCl_3_ radicals [55,56]. Therefore, consumption of its stores by CCl_4_-induced scavenging free radicals is indicative of liver injury [57]. Glutathione peroxidase (GPX) plays a pivotal role in protective defense system in the liver [58]. Diminished activity of GPX, due to ROS, is also another manifestation of propagated liver injury.

**Figure 7:**
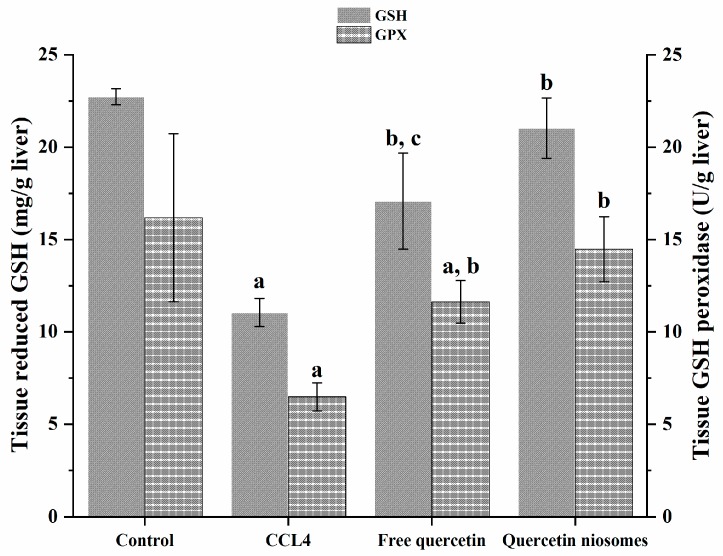
Effect of pretreatment with free quercetin and quercetin niosomes on GSH level and GPX activity. Notes: a = significantly different from control group; b = significantly different from CCl_4_ group; c = significantly different from quercetin niosomes.

Marked depletions of both non-enzymatic and enzymatic sides of defense mechanism in liver tissue were observed in rats intoxicated with CCl_4_. As shown in Figure 7, there was a significant decrease in the level of reduced glutathione (GSH), as well as the activity of antioxidant enzyme, and glutathione peroxides (GPX) by 51.40% and 59.95%, respectively, was noticed, comparable to those of the normal control group (*p* < 0.05). In contrast, rats pretreated with quercetin, both in free and loaded forms, showed a significant change in oxidative stress markers, in terms of restoring GSH level and GPX activity, in comparison to the CCl_4_ deleterious toxicity (*p* < 0.05). However, the antioxidant potential of quercetin was more pronounced in quercetin niosomes, greatly alleviating the free radical damage via amending the oxidative stress markers to almost the normal level compared to free drug (*p* < 0.05). These results greatly indicated the potential of the nanocarriers in enhancing cellular uptake.

#### 3.8.3. Histopathology

**Figure 8:**
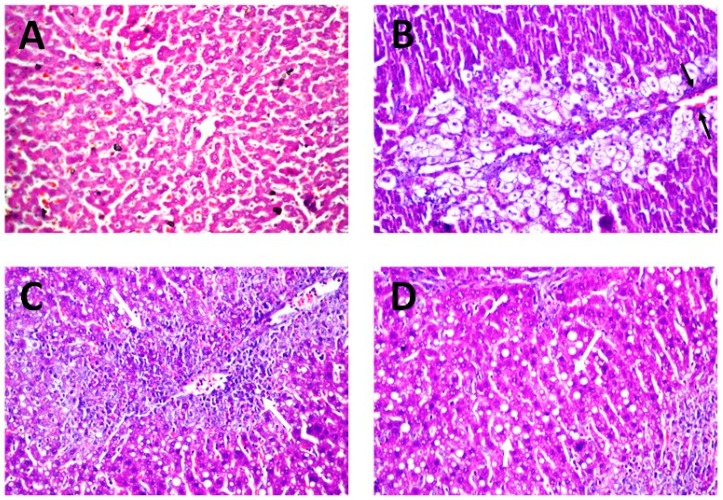
Histology of liver tissue autopsy of rats, using microscope magnification 40×, after administration of (**A**) normal saline (negative control), (**B**) CCl_4_ (positive control) and (**C**,**D**) quercetin niosomes.

The serum markers of hepatic injury were further endorsed by the results of histopathological investigation. As revealed in Figure 8A, photomicrograph liver biopsies after administration of the normal saline (negative control) exhibited no histoarchitecture alteration. The normal structure of the central vein and surrounding hepatocytes in the parenchyma, with well-preserved cytoplasmic and nuclear appearance, were recorded. On the other hand, photomicrograph of rats intoxicates with CCl_4_ depicted remarkable centrilobular necrosis and ballooning degeneration in the hepatocytes surrounding the central vein in diffuse manner all over the hepatic parenchyma. Considerable congestion of blood vessels and fatty globules accumulations were also detected, as denoted by the black arrows in Figure 8B.

Promising protective activity of quercetin niosomes was determined as evidenced from the amelioration of these histopathological distortions. The hepatocytes surrounding and adjacent to the central vein showed necrobiotic changes and fatty change in parenchyma, as denoted by white arrows, markedly reversing the CCl_4_ intoxication during the peak period (Figure 8C,D). Similar protective efficacy was confirmed by using natural phytochemicals [59].

## 4. Conclusions

By combining hepatoprotective activity of natural flavonoid quercetin and drug delivery potential of niosomes, in this work, we have developed and characterized different quercetin-loaded niosomal formulations, using monolaurate sugar esters (i.e., sorbitan C12, glucose C12, trehalose C12 and sucrose C12). Among the prepared batches, F2 formula, containing glucose C12 and cholesterol in a molar ratio of 1:1, presented a PS of 161.0 ± 4.61 nm, round shape, a very uniform dispersity and an EE% of 83.59 ± 3.66%, with a diffusion-controlled release mechanism. Thanks to these characteristics, this formulation was chosen for in vitro and in vivo investigations, presenting low cytotoxicity at concentrations up to 20 μg/mL of encapsulated drug.

The hepatoprotective effect was demonstrated first in vitro on HepG2 cells and then in vivo on rats, with CCl_4_-induced hepatic toxicity. Serum biomarker enzymes ALT and AST significantly decrease after pretreatment with quercetin niosomes, as well as ALP and total protein in serum.

Pretreatment with quercetin niosomes also significantly helped in restoring the GSH level and GPX activity.

Together, these results confirm the potential hepatoprotective effect of the quercetin-loaded niosomal formulation increasing the activity of the free natural flavonoid quercetin. Moreover, the nanosized system has been prepared by using the fast and easy ethanol-injection method, which is potentially scalable for larger production.

## Figures and Tables

**Table 1:** Selected surfactant for niosomes formulation.

Surfactant (C12 Chain)	HLB ^a^	Mw
Sorbitan monolaurate (Span^®^ 20)	8.6	346.5
Glucose monolaurate	9.89	362.5
Sucrose monolaurate	13.01	524.6
Trehalose monolaurate	13.01	524.6

^a^ Calculated HLB by Griffin’s method for non-ionic surfactants. (HLB = 20 × (MW hydrophilic portion/MW)) [32].

**Table 2:** Composition and characteristics of prepared niosomal formulations.

Formula Code	Surfactant Type	Surfactant: Cholesterol Ratio	PS (nm)	PDI	ZP (mV)	EE%
F1	Sorbitan C12 (Span^®^ 20)	1:1	177.4 ± 1.89	0.19 ± 0.07	−26.23 ± 1.56	80.85 ± 2.00
F2	Glucose C12	1:1	161.0 ± 4.61	0.09 ± 0.01	−28.75 ± 0.07	83.59 ± 3.66
F3	Sucrose C12	1:1	179.0 ± 2.99	0.24 ± 0.02	−32.2 ± 0.42	54.60 ± 2.72
F4	Trehalose C12	1:1	174.1 ± 9.28	0.25 ± 0.03	−25.25 ± 0.07	62.13 ± 2.64
F5	Sorbitan C12 (Span^®^ 20)	2:1	188.9 ± 3.61	0.11 ± 0.03	−27.54 ± 2.35	55.44 ± 1.30
F6	Glucose C12	2:1	217.4 ± 3.52	0.12 ± 0.08	−25.3 ± 3.53	61.26 ± 2.21
F7	Sucrose C12	2:1	224.4 ± 4.38	0.34 ± 0.04	−35.25 ± 1.20	56.15 ± 1.49
F8	Trehalose C12	2:1	222.9 ± 8.74	0.26 ± 0.02	−33.05 ± 1.22	62.14 ± 5.63

PS: particle size; PDI: polydispersity index; ZP: zeta potential; EE%: encapsulation efficiency.

**Table 3:** Measured parameters of rats with CCl_4_-induced hepatotoxicity.

		Normal Untreated Control	Positive CCl_4_ Treated Control	Free Quercetin	Quercetin Niosomes
Serum biomarker enzymes	ALT (IU/L)	37.5 ± 1.5	164.78 * ± 5.19	94.93 ± 7.35	63.80 *^,+^ ± 7.52
	AST (IU/L)	34.07 ± 0.76	136.52 * ± 1.23	85.02 ± 2.04	69.49 *^,+^ ± 6.02
Serum biochemical parameters	ALP (IU/L)	118.38 ± 3.13	191.37 * ± 3.5	138.24 ± 2.68	128.57 *^,+^ ± 4.29
	Total protein (g/dL)	6.84 ± 0.07	7.63 * ± 0.27	6.59 ± 0.15	6.71 *^,+^ ± 0.06

Data are given as the mean ± SEM (*n* = 6); * *p* < 0.05 vs. normal untreated control; ^+^ *p* < 0.05 vs. positive CCl_4_-treated control.
